# Histological and Electrophoretic Analysis of Carpathian barbel (*Barbus carpathicus*, Cyprinidae) Skin and Mucus in Environmental Context

**DOI:** 10.3390/ani10040645

**Published:** 2020-04-08

**Authors:** Przemysław Spychalski, Dominik Poradowski, Aleksander Chrószcz

**Affiliations:** Division of Animal Anatomy, Department of Biostructure and Animal Physiology, Faculty of Veterinary Medicine, Wroclaw University of Environmental and Life Sciences, Kożuchowska 1, 51-631 Wrocław, Poland; bjorn@onet.pl (P.S.); aleksander.chroszcz@upwr.edu.pl (A.C.)

**Keywords:** barbel, common integument, electrophoresis, fish, histology, seasons

## Abstract

**Simple Summary:**

Natural environment monitoring and identification of river or lake water pollution plays an important role for human comfort and safety. Some fish, as water-living animals, are especially sensitive to any water quality changes. Fish skin forms a natural border between the water and internal organs of the animal’s body; therefore, the skin must be able to provide defense and resistance against any factors dangerous for the fish. Some seasonal changes can influence the skin morphology and physiology (mucus composition), blurring the assessment of the skin’s reaction to any contamination.

**Abstract:**

Fish frequently serve as bioindicators of aquatic environments during their ecological evaluation. Carpathian barbel (*Barbus carpathicus*, Cyprinidae) is a species common to rivers and lakes of Eurasia and Africa. Seasons of the year can influence its skin morphology and mucus composition. The clinical status of the animal depends on the above-mentioned factors. The aim of this study was a histological, histometrical and electrophoretical analysis of periodical changes in barbel common integument. The accessible material was investigated in histological, cytological and electrophoretic analysis using hematoxylin-eosin staining, histometric morphometry, gel electrophoresis and cytological methods. The results demonstrated significant differences in the investigated parameters for spring–summer and autumn–winter periods. Both skin epithelium morphology (epithelium thickness, number of cell layers, melanophores and mucous cell existence) and mucus composition (proteins, immune system cells, keratinocytes and mucocytes) showed significant differences between investigated seasons. These morphological and physiological changes were more pronounced in the dorsal than ventral regions of common integument. The differences in the physical characteristics of mucus and the histological structure of the skin cannot only serve as a source of useful information about an evaluated ecosystem, but can be also related to additional factors, e.g., microbiological and chemical water contamination.

## 1. Introduction

One of the features of living organisms is their ability to adapt to variable environmental conditions. Any organism’s existence is limited by two extreme values of a specific factor, i.e., its minimum and maximum. The ability of living organisms to adapt to changing environmental conditions is called tolerance, and its range may by narrow in stenobionts or widen in eurybionts. Species with narrow tolerance ranges to specific environmental conditions serve as indicator species, or bioindicators [1,2].

The numerous plant and animal species used as pollution bioindicators include algae (cyanobacteria and green algae in eutrophicated waters of poly- and alphamesosaprobic zones), diatoms (nonhazardous water pollution of betameso- and oligosaprobic zones), euglenoids (zones of strong and medium water pollution), red algae, sponges, tubellaria, mayflies, caddisflies, crustaceans (clean waters), flagellates and ciliates (polluted waters), annelids, mollusks and insects (used to determine the degree of soil and water pollution), and amphibians, fish or mammals (indicators of water, air and soil pollution) [3,4].

The barbel is a genus of carp-like freshwater fish belonging to the family of Cyprinidae, comprising a large number of species. A typical species of this genus is *Barbus barbus* (Linnaeus, 1758). Systematics of the genus are heterogeneous and, for example, populations of *Barbus petenyii* were identified to include three separate species—*B. petenyii*, *B. carpathicus* and *B. balcanicus* [5,6]. Contrary to other cyprinids, the barbel’s body is elongated, cigar-shaped and nonarched. The upper lip has two pairs of barbels, the eyes point slightly upwards, the dorsal and anal fins are short, and the caudal fin is longer and forked. The fish coloration ranges from olive green to dark cream. Its distinctive feature is bright-red fins, except for the dorsal and caudal fins. The average body length is 70 cm, the but maximum length is up to 120 cm and the body weight may then reach 12 kg. The species feeds on larvae of aquatic insects, mollusks, oligochaetes and rarely, small fishes [6]. Spawning takes place between May and August at 15–18 °C, usually in shallow water with a sandy and stony bottom. Males have a visible spawning rash on their backs and heads. Barbel roe is poisonous [7,8]. Since 2008, Carpathian barbel has been present on the Red List of Threatened Species (IUCN).

Apart from its great economic importance, the fish is also used as a bioindicator in water treatment processes mainly due to its high sensitivity to changes in water oxygen levels. The phenomenon of gill remodeling in some Cyprinidae is well-known. Nilsson [9] proved that hypoxia causes interlamellar cell mass reduction in order to increase the surface of oxygen intake in crucian carp, goldfish and mangrove killifish. This adaptive reaction present in the closely related fish mentioned above suggests that similar changes can occur also in barbel. A water temperature increase results in lower oxygen concentrations in the water. This fact can also explain changes in the morphology of gills. Schaack and Chapman [10] describe the correlation between water hypoxia, animal behavior and food intake. The feeding process in modern fish farming plays an especially important role due to the economy of animal production. Methods to assess the quality of river water vary greatly in individual European countries. Almost each country uses its own water purity assessment system [11,12]. The system currently in operation in Poland is a saprobic one. It evaluates a degree of organic contamination of water based on a biological analysis of the biocenosis composition, and qualitative and quantitative changes in this composition, in the presence of contamination. The saprobic system is based on the tolerance of indicator species belonging to many groups, e.g., bacteria, algae, protozoa and rotifers, and sometimes also vascular plants and fishes [11]. The World Health Organization (WHO) recommends using fish highly sensitive to pollution when assessing water condition.

The aim of this study was to determine the morphological changes of Carpathian barbel skin, cytological profile and protein content of its mucus, depending on the season and reproduction period. We also intended to assess the possibilities of using the species as a bioindicator to monitor the environmental conditions of aquatic bodies and watercourses.

## 2. Materials and Methods 

The study material included 40 individuals. They were obtained from a fish farm in “Szczodre” (Polish Angling Association), Lower Silesia, Poland. The animals were harvested four times—in the spring, summer, autumn and winter.

Whole material was accessible and the tissues were collected during other investigations. According to the Polish legacy system, Ethic Committee acceptance is not needed for tissue collection.

First, mucus was collected from their skin and subjected to analysis. The mucus was collected by hand, "caressing" the fish gently in the caudal direction, so as not to cause injury to scales. The mucus was not thinned or centrifuged prior to separation into individual groups.

**Figure 1:**
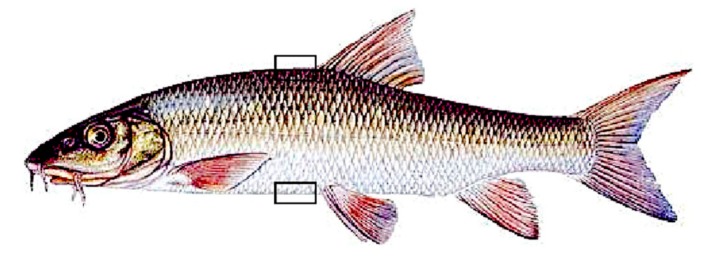
Location of sample collection.

Then, skin specimens were taken from each individual from dorsal and ventral regions. The samples were collected at the level of the pectoral fin, ca. 1 cm caudally from its posterior edge and cranially from the dorsal fin (Figure 1).

### 2.1. Histological and Histometrical Analysis

Histological specimens were fixed in a 4% solution of calcium carbonate-buffered formalin at pH 7.0 for three days and then rinsed in running water for 24 hours. Then, the material was dewatered using a standard procedure of alcohol series, brightened in a solution of methyl benzoate, and finally embedded in paraffin. Histological sections, 7 µm thick, were dewaxed, hydrated and stained with hematoxylin and eosin (H-E). After drying, the histological slides were covered with cover slips and Canadian balm.

The histological and histometrical analysis was performed under a light microscope Nikon Eclipse 80i (Japan) in transmitted and polarized light. Morphometric examination of the epidermis was performed with Nis Elements Ar software. The histometrical analysis covered not only the thickness of the epidermis and the presence of mucus cells, but also the number of epidermis layers. All measurements were taken with calibrated histometric software at 10 measurement points. Each measurement (from basal membrane to epithelium surface, perpendicular to the epithelium surface) was made three times and subsequently the mean value was computed. Statistical analysis was performed in Statistica PL software (StatSoft Polska, Kraków, Poland).

### 2.2. Electrophoretic Separation of Proteins

The tests involved electrophoresis in a 10% polyacrylamide gel considered optimal for this type of sample. The most commonly used polymerization catalyst is polytetramethylethylenediamine (TEMED), (CH3) 2NCH2CH2N(CH3)2’ coupled with (NH4)zS20S ammonium persulphate (APS) as an initiator, and the process consists of the formation of two stable free radicals, each with an unpaired electron.

Protein electrophoresis for mucus investigation was performed in 10% (w/v) polyacrylamide gel (gel composition provided in Table 1). The following buffers were used:

a) Stacking gel buffer: 0.5M Tris-HCl pH 6.8 with 0.4% SDS (sodium dodecyl sulfate); and
b) Separating gel buffer: 1.5M Tris-HCl pH 8.8 with 0.4% SDS.

The separating gel (10%) was poured in between two glass plates previously degreased and rinsed in acetone, and then 4% stacking gel was prepared. The investigated material was loaded after 1 h.

The mucus samples (10 µl) were loaded into wells. They were diluted 1:20 after five minutes of boiling at 100 °C in the sample buffer containing 0.5 M Tris-HCl, glycerin, SDS (37.5:30:6), and bromophenol blue 0.2 g/l, pH 6.8. The separation was performed in a vertical Bio-Rad Mini-Protean 3 system in an electric field at a constant voltage of 80 V (voltage gradient about 13.3 V/cm) in the stacking gel and 120 V (voltage gradient about 20 V/cm) in the separating gel. The electrode buffer consisted of 0.5 M Tris, 0.192 M glycine, and 0.1% SDS, pH 8.3. The electrophoresis was stopped when bromophenol blue reached the bottom of the gel.

Following separation, the proteins were visualized with Coomassie blue in a three-stage staining procedure:

1) Fixing (10 min) in a mixture of methanol, glacial acetic acid and distilled water (50:9:41);
2) Staining at room temperature, with Coomassie blue 0.5 g/l; and
3) Discoloration until a colorless background was reached with a mixture of methanol, glacial acetic acid and distilled water (25:7:68).

Then, the samples were analyzed by comparing their molecular weight with that of the standard proteins provided in kDa: 90, 67, 60, 30, 20 and 14. The following scale was used to quantify the proteins: (+) weak staining, (++) moderate staining and (+++) strong staining. The staining intensity was always evaluated by the same person. All results are presented in the form of figures and tables.

## 3. Results

**Figure 2:**
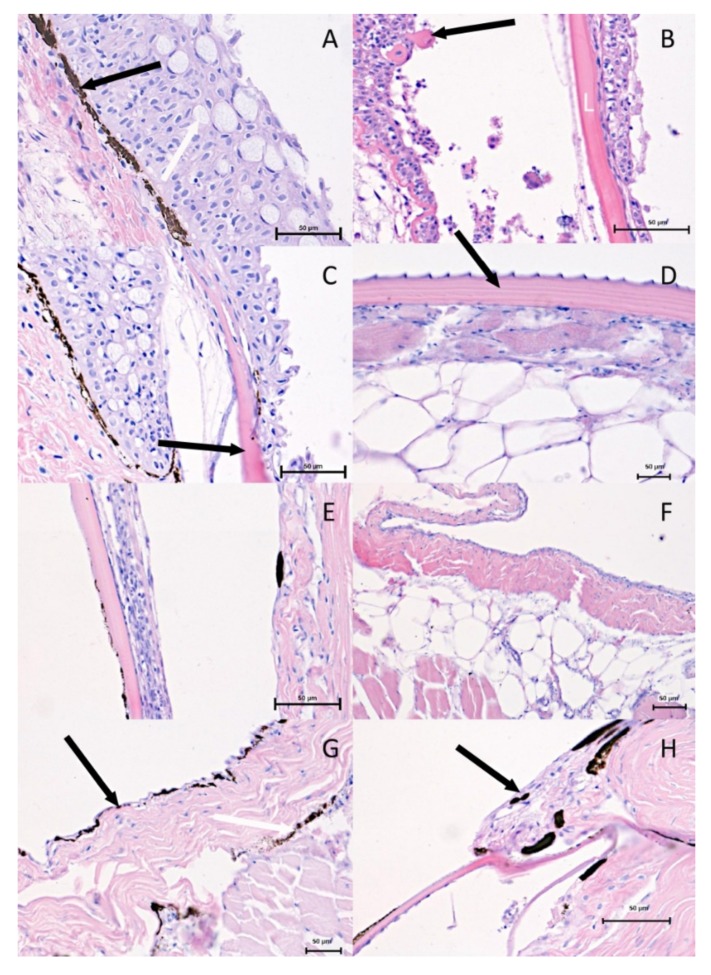
Skin in the spring-summer period: **A**. The dorsal area; numerous melanophores are visible (arrow) directly under the basal layer of the epithelium. The surface layer contains numerous mucus cells (white arrow). H-E 400x. **B**. The ventral area; the epithelium visible on a scale (L) with single, developing forms of mucus cells. No melanophores within this area. Clusters of fat cells and alarm cells (arrow) visible under poorly developed connective tissue. H-E 400x. **C**. The dorsal area; the epithelium covering a scale (arrow) with drastically reduced number of layers. No melanophores within this area. H-E 400x. The skin in the autumn-winter period: **D**. The ventral area; the scale (arrow) not covered with the epithelium. The scale adheres directly to the connective tissue underlined with adipose tissue. H-E 400x. **E**. The dorsal area; delaminated stratified epithelium with small mucus cells covered by a scale. H-E 200x. **F**. The ventral area; the skin is covered with the epithelium with few cell layers without mucus cells. Well-developed dermis made of collagen fibres. Clusters of yellow adipose tissue in the hypodermis between the dermis and muscles. H-E 400x. **G**. The dorsal area; strong local reduction of epithelium (arrow) seemingly in contact with melanophores. The second melanophore layer on the border of dermis and hypodermis (white arrow). H-E 200x. **H**. The dorsal area; strong local reduction of epithelium (arrow) near a scale. Melanophores remain in the skin fold accompanying the scale. H-E 400x.

The histological observations using a light microscope are shown in Figure 2. The histological images taken from the dorsal and ventral region in both investigated periods show significant differences. The epithelial thickness and the numbers of cell layers in the spring-summer period (Figure 2A–C) are greater than in the autumn-winter period (Figure 2D,F,G,H).

**Figure 3:**
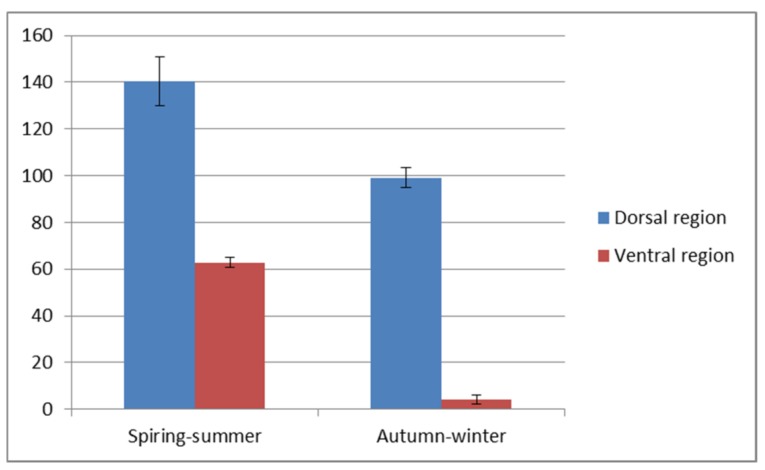
Epithelium thickness (µm) in the dorsal and ventral skin of the investigated fish.

**Figure 4:**
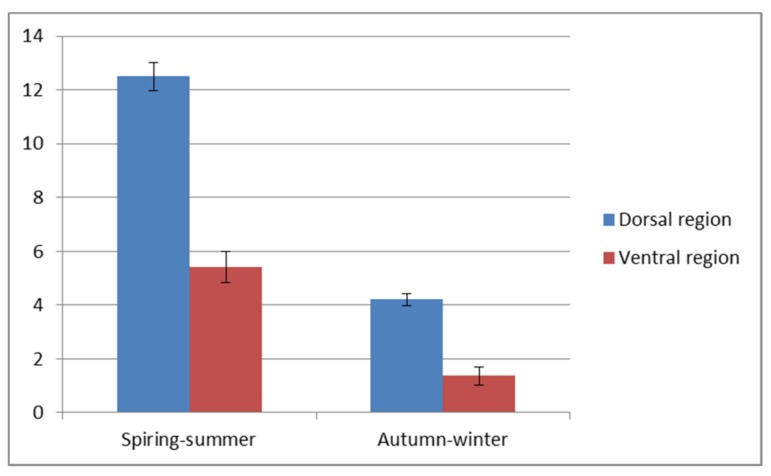
Number of epithelial layers in the fish skin.

A visible reduction of the epithelial thickness in the autumn-winter period was stronger in the ventral than in the dorsal region of the trunk. The detailed values of both parameters are shown in Figure 3 and Figure 4. The significant differences (*p* < 0.5) are stated for the dorsal and ventral region of the trunk. The thicker, dorsal region had from eight to sixteen layers of cells and two to five layers in thinner ventral region.

Directly under the basal membrane, a greater number of melanophores was visible in the dorsal region (Figure 2A) in the spring–summer period. The only exception is the epithelium, which directly covers the scale. The mentioned epithelium was also strongly reduced (Figure 2C). On the other hand, the presence of melanophores in the ventral region was not proven in the spring–summer period (Figure 2B). In the autumn-winter period, the most significant differences were observed between the dorsal and ventral regions. The melanophores formed two layers in the dorsal region (Figure 2G), and were also seen in the region of the scale (Figure 2H), in contrast to the ventral region where no melanophores were observed (Figure 2D,F).

**Figure 5:**
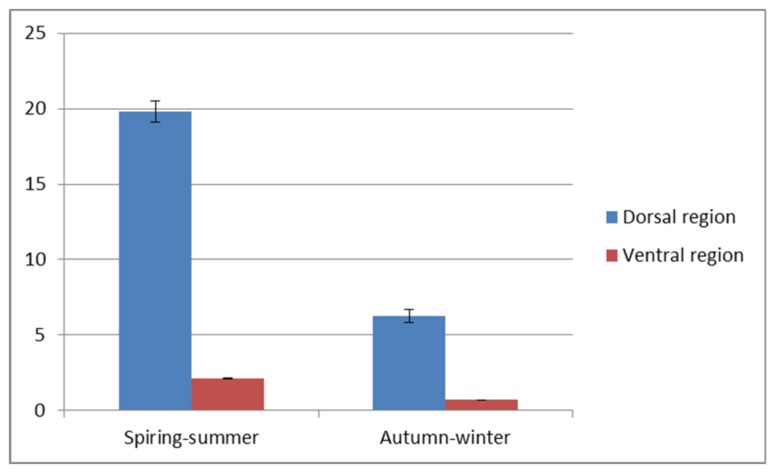
Presence of mucus cells (%) in the fish skin in the investigated periods.

Similar changes were visible in a histological picture of skin glands, the abundance of which was higher in the spring-summer period (Figure 2A,C) and lower in autumn-winter (Figure 2E,G,H). The number of mucous cells was greater in the dorsal region in the spring-summer period (Figure 2A,C). In the same region in the autumn-winter period (Figure 2E,G,H) and in both periods in the ventral region, the mucous cells were less numerous (Figure 2B,D,F). The presence of mucous cells (Figure 5) in both of the above-mentioned time periods was also proved to be statistically different.

The connective tissue of the dermis was thin (Figure 2F).

**Figure 6:**
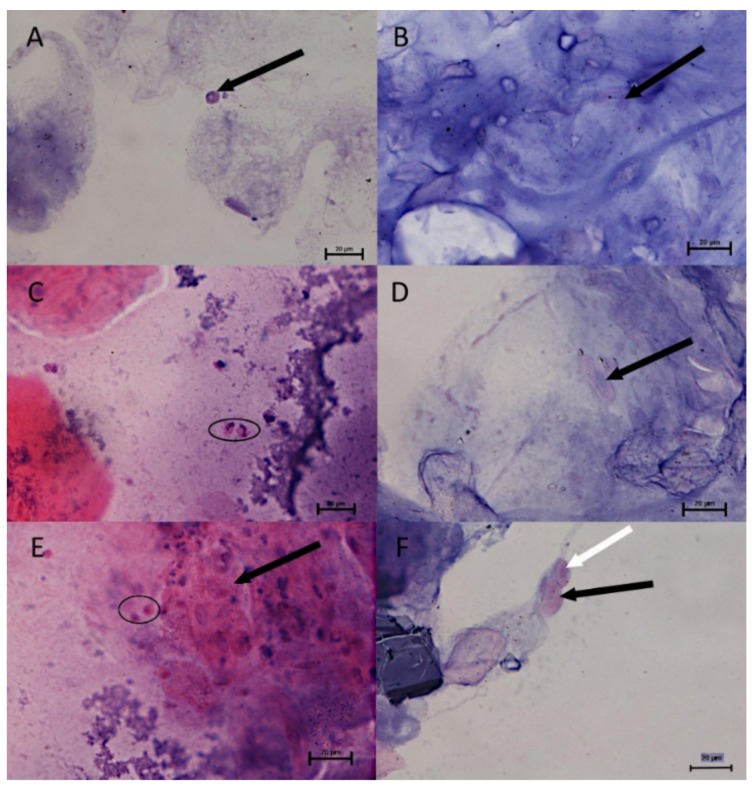
Cytological pictures of mucus: **A.** Mucus from the autumn-winter period; with a single immune cell (a macrophage, arrow). H-E 600x. **B.** Mucus from the spring-summer period; with lymphocytes (arrow). H-E 600x. **C.** Mucus from the autumn-winter period; with some neutrophils (circle). H-E 600x. **D.** Mucus from the spring-summer period; with visible interconnected epithelial cells (arrow). H-E 600x. **E.** Mucus from the autumn-winter period; with macrophages (circle) and exfoliated epithelium cells (arrow). H-E 600x. **F.** With a keratinocyte (arrow) and a migrating phagocyte (white arrow). H-E 600x.

**Figure 7:**
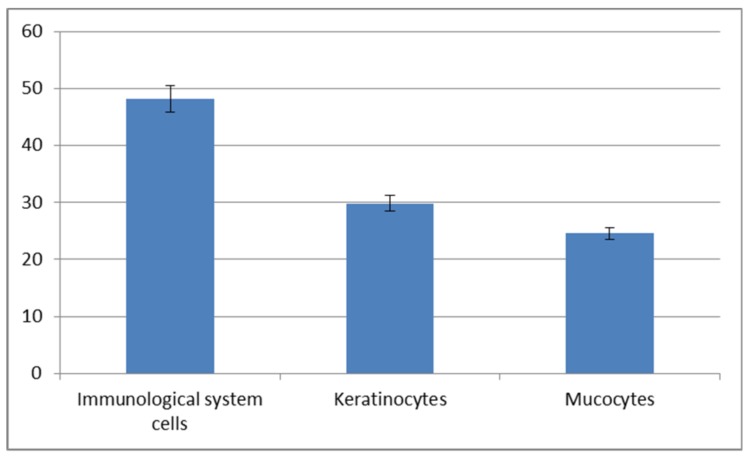
Cellular composition of mucus collected in the autumn-winter period.

**Figure 8:**
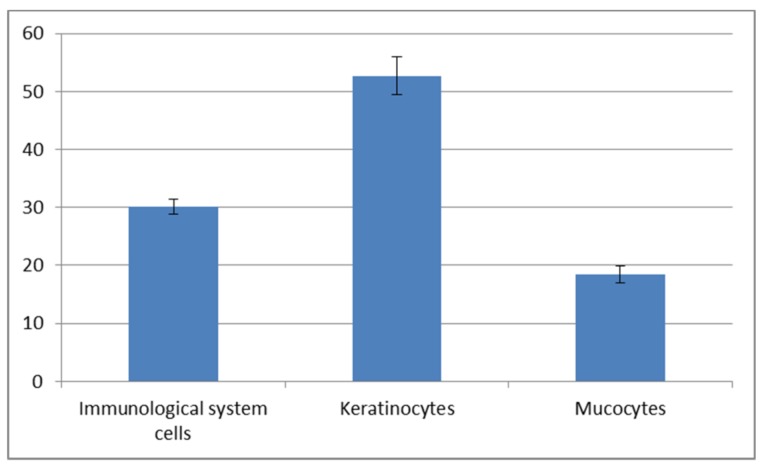
Cellular composition of mucus collected in the spring-summer period.

**Figure 9:**
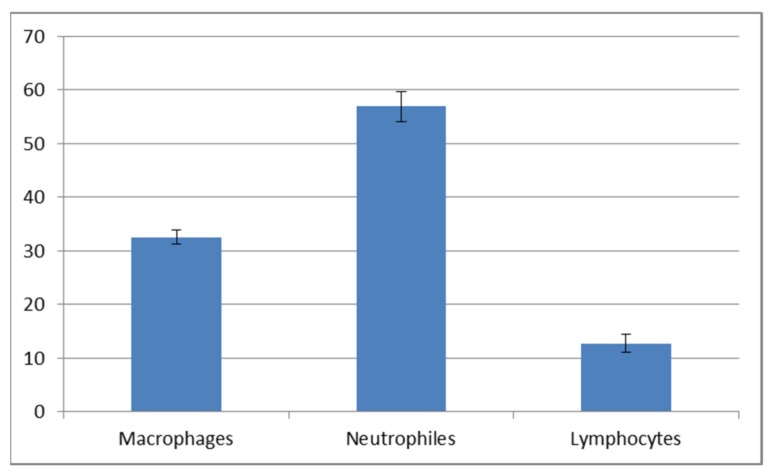
Percentage share of immune cells in the autumn-winter mucus.

**Figure 10:**
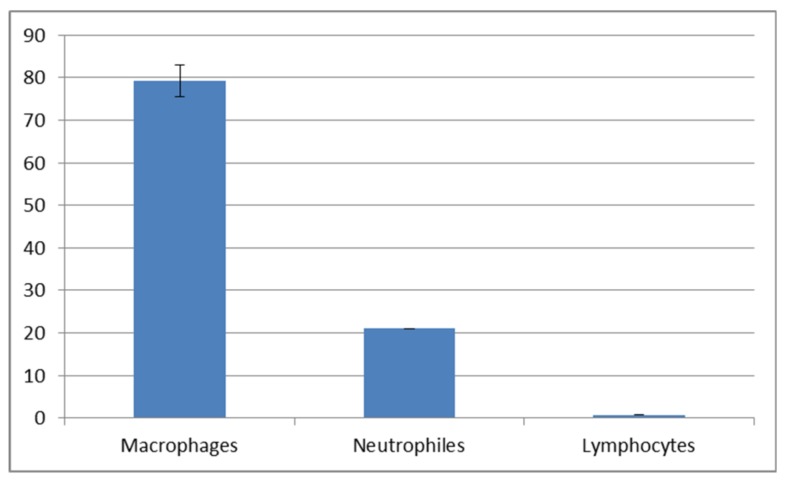
Percentage share of immune cells in the spring-summer mucus.

The cytological observations are presented in Figure 6. The epithelial and immune system cells (macrophages, neutrophils and lymphocytes) were present in the mucus volume. The differences between the spring-summer and autumn-winter periods consist of the presence of keratinocytes and phagocytes in mucus, accompanied with delaminated epithelial cells and lymphocytes (Figure 6B,D,F) in the first-mentioned period and the macrophages, neutrophils and epithelial cells observed in the second period (Figure 6A,C,E). The statistically significant increase of immune cells present in the autumn-winter period contrasts with the higher quantity of keratinocytes in the spring-summer period (Figure 7 and Figure 8). Similar findings were made from the analysis of immunological system cell intake differences (Figure 9 and Figure 10). The macrophage percentage in mucus from the spring-summer period was higher than the neutrophil intake in the same period. In the autumn-winter period, the presence of the above-mentioned cell types was reversed.

**Figure 11:**
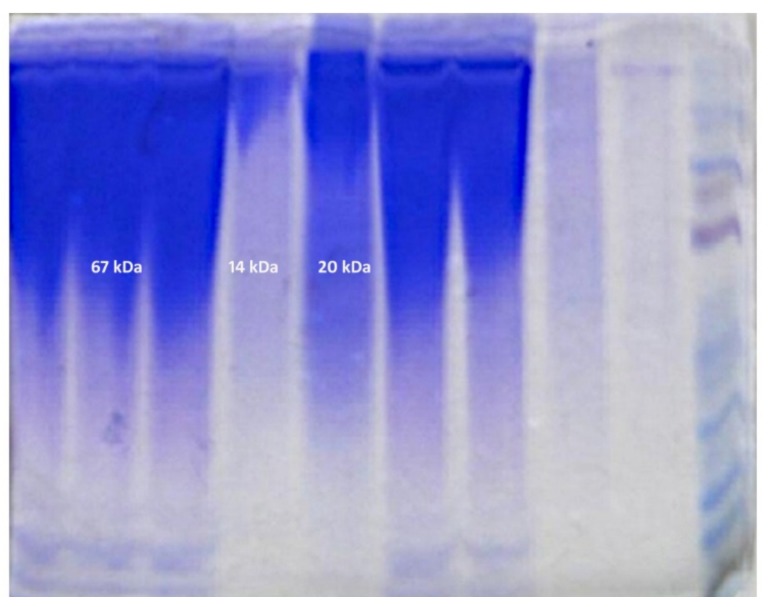
Electropherogram of the investigated protein samples in the spring-summer period.

**Figure 12:**
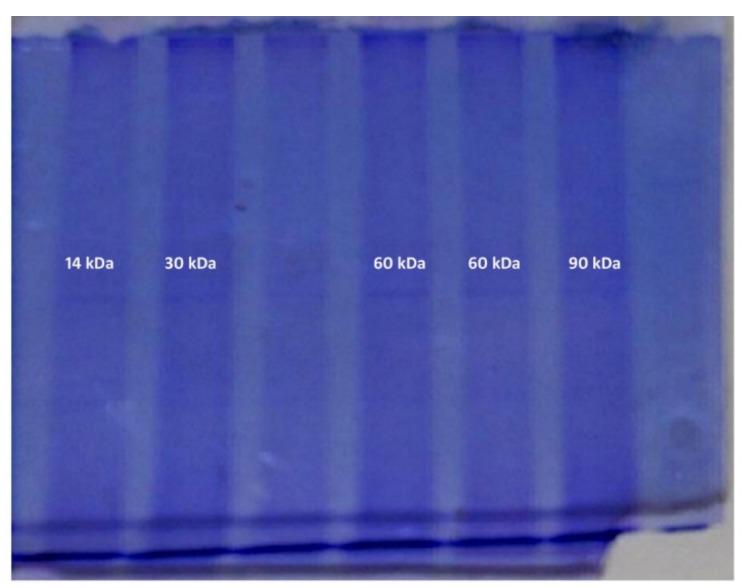
Electropherogram of the investigated protein samples in the autumn-winter period.

The electrophoretic analysis proved the qualitative differences in protein composition in mucus (Figure 11 and Figure 12). The stacking and separating gel composition is shown in Table 1. The molecular protein weights (kDa) detected in the spring and summer were 14, 20 and 67 kDa, and in the autumn and winter were 14, 30, 60 and 90 kDa (Table 2).

## 4. Discussion

The present histological and morphometrical study examined the skin of *Barbus carpathicus*. Due to small variability of the common integument morphology between autumn and winter, and spring and summer, the seasons were pooled together into autumn–winter and spring–summer periods.

The skin structure of the barbel, similar to other cyprinids, shows considerable structural differences depending on the body area. The fish skin comprises two types of tissue—epithelial (textus epithelialis) and connective (textus connectvus). The first is superficial and serves as a direct mechanical barrier between the external environment and the animal body, and the second is a component of deeper layers of the common integument. The typical skin structure is comprised of the epidermis (epidermis), dermis (cutis propria) and hypodermis (hypodermis). The hypodermis connects the remaining skin layers with deeper tissues (muscles and skeleton components) [13,14]. Specific skin structures allow for the maintenance of homeostasis in the fish body. In fish, the superficial epidermal cells in contact with water form a cuticle. They peel off and are replaced by the cells of the next layer [15,16]. A typical feature of the stratified fish epidermis is an almost complete lack of keratin. The dermis is considerably reduced, and the epidermis surface is protected by scales and mucus [17].

The skin of the teleosts (Teleostei) contains numerous single-cell mucus and serous glands. Clusters of those glands (secreting proteases) are located at the anterior pole of the head. Similar to elasmobranchii and some teleosts, e.g., trout and crucian carps, these glands are used to open egg envelopes. They should not be confused with cement glands in the cyprinids and pikes that allow the newly hatched fry to attach to aquatic plants [13,14]. Similar to other cyprinids, Carpathian barbel skin in the dorsal region is thicker and comprises between eight and sixteen layers of cells. The gland abundance grows in the spring and summer period (Figure 2A,C) and declines in the autumn and winter (Figure 2E,G,H). The changes are due to, e.g., increased number of mucus-secreting cells in this area of the common integument. The period of intense epithelial proliferation is relatively short and therefore the number of its layers does not increase to a considerable degree. The mucus cells are interspersed with single migrating cells identified in a cytological examination of the mucus covering the fish body.

Contrary to the back, epithelium thickness decreases towards lateral-ventral regions, about 1 cm caudally from the pectoral fin, where it forms only two to five layers of cells. Locally, on the scales and ventral region, the epithelium is extremely thin. In winter, epithelial cells are seemingly in direct contact with melanophores (Figure 2G). In the ventral region, there are not many differences for the investigated periods, and variability in this body region is the lowest (Figure 2B,D,F). It lacks mucus cells, particularly during autumn and winter, while single cells may appear in the spring and summer. Epithelial cells are easily separated by mechanical factors, which is why mucus plays an important role in preventing their excessive exfoliation. At some spots, delamination of larger epithelium fragments was visible and the epithelium became thinner than in the neighboring areas. Clusters of epithelial cells can be found in the mucus much more often in the spring-summer than the autumn-winter period.

Just under the epithelium, melanophores (pigment cells) can be seen in the dorsal and lateral regions (Figure 2A,C). They do not appear on the ventral region of the common integument (Figure 2B,D). The melanophores are arranged in layers and form membranes. The first layer is located directly under the epithelium, and the second, common in many species, in deeper layers of the dermis. The third layer occurs only in the apical region of the back and reaches deep into the subcutaneous layer. The dermis connective tissue is thin (Figure 2F), and nearly directly contacts skeletal muscles. In some individuals the dermis is separated from the skeletal muscles by a well-developed layer of subcutaneous fat. Apart from the mucus cells in the epithelium, these regions of skin contain no other glands. In the spring and summer, the ventral epithelium incorporates acidophilic, pear-shaped, mononuclear alarm cells, the secretion of which is part of the mucus and intensifies under stressful conditions (Figure 2B).

The surface skin layers incorporate areas responsible for scale formation that are covered with a skin fold with stratified epithelium containing mucus cells. Furthermore, also in this case, melanophores occur just below the epithelium. The skin fold epithelium may partially cover a scale in the beginning, but it rapidly flattens at a distance of several dozen µm from the scale base. A pocket from which a scale emerges from the skin in the spring and summer is lined with a simple squamous epithelium (Figure 2A,C). In the autumn and winter, the epithelium thickness sharply decreases (Figure 2E,G,H), and growth of the scales is inhibited. In this period, the common integument is particularly sensitive to mechanical injury that may potentially cause an infection. The reduced amount of mucus in the autumn-winter period is a disadvantageous phenomenon as it hinders the fish’s ability to maintain the osmotic balance between its body and the surrounding environment.

Our morphometrical analysis showed significant differences (*p* < 0.5) in the epithelium thickness in the autumn-winter and spring-summer periods between the dorsal and ventral skin regions (Figure 6A,C,E). These were due to a changing number of mucus cells and epithelial layers. In the autumn and winter, the number of mitotic divisions in the skin is lower, thus affecting the skin’s regenerative abilities. Variations in the dorsal epithelium thickness to the greatest extent depend on the presence of scales that locally may be closely fused to the epithelium, particularly in the autumn-winter period. The number of epithelial layers enhances caudally, and the arrangement of adjacent scales further increases the thickness of the skin.

However, mucus secreted by the epithelial mucus cells still plays the most important role in reducing water resistance. The secretion is very slippery, it eliminates friction and its transparency does not significantly affect masking coloration of the fish. Fish mucus may differ in its physical and chemical properties and function. In some species it may show bactericidal, antifungal and antiparasitic activity. It may contain lysozymes, interferons or trypsin. Mucus also facilitates migration of keratinocytes towards the surface of skin that has experienced mechanical damage, thus playing an important role in repair and regeneration processes. Interestingly, most wounds in the fish common integument heal without scarring.

Figure 6, Figure 7 and Figure 8 show the results of our cytological analysis. The mucus cells included delaminated single or clustered epithelial cells and immune system cells (usually macrophages and neutrophils or lymphocytes). We also detected exfoliated mucus cells or their residues.

Our study demonstrated an increased percentage share of immune system cells (particularly macrophages) in the mucus in the autumn–winter period and keratinocytes in the spring and summer months. The reason for this is a decline in the number of mitotic divisions of the epithelial cells and a resulting decrease in the delamination rate. The presence of immune cells followed a slightly different pattern. In the spring and summer, the most commonly spotted cells were macrophages (78%) while, in the autumn and winter, we mostly noted neutrophils accompanied by an increase in lymphocytes and a decline in macrophages (down to 32%) (Figure 9 and Figure 10). The changing share of immune system cells in the investigated mucus may reflect variable activities of the system. Macrophages are responsible for phagocytosis and presentation of antigens to the other cells of the immune system. Neutrophils use their hydrolytic enzymes to degrade bacterial and fungal pathogens, damaged cells and foreign bodies. The presence of lymphocytes and plasma cells may indicate an ongoing inflammatory process. Cytotoxic lymphocytes are responsible for destroying bacterial cells and foreign cells (including cancer cells), while plasma cells synthesize antibodies against specific antigens.

The investigated material showed considerable quantitative and qualitative differences depending on the season (Table 2). The mucus collected in the autumn–winter season contained proteins of larger molecular weight than that collected in the spring and summer. For these periods we also discovered morphological and morphometrical differences in the skin. Protein composition analysis of the fish mucus performed for both periods revealed variations in their molecular weight (Figure 6B,D). Considering the work of Iwama et al. [18], it should be noted that proteins detected in the autumn and winter are associated with the activity of immune cells and protect the epithelial cells against mechanical damage and infections [19,20]. In the spring and summer, the proteins not only support the animal’s resistance, but also protect them against pollution (zinc 22).

Molecular weights (MWs) of the proteins detected in the autumn and winter were 14, 30, 60 and 90 kDa. The protein with a molecular weight of 90 kDa was described in fish by W. J. Cho [19] and classified as a protein appearing in response to rhabdovirus infection. The 60 kDa protein is a cell membrane protein associated with inflammation and an immune response [18,20]. This hypothesis is also supported by the presence of lymphocytes and plasma cells found in the mucus of the investigated animals. The 30 kDa protein is a Heat Shock Protein (HSP), the synthesis of which is triggered by changes in ambient temperature. HSPs protect the functional proteins against changes in their spatial conformation [17,21,22]. Their presence is related to the body response, including nervous and endocrine systems, to long-term increase or decrease of the ambient temperature [23,24,25,26,27]. The 14 kDa protein was detected throughout the year [28], and is synthesized in response to heavy metal contamination, particularly with zinc and cobalt oxides. Water filtering devices at the Polish Angling Association “Szczodre” fish farm and containers for fish storage before transport are made of zinc sheets. This may explain the presence of these proteins in the mucus.

An additional protein of MW 67 kDa appeared in the spring and summer in response to water pollution with the above-mentioned chemicals. In the autumn–winter period, water temperature is lower and the content of metal oxides in the reservoirs is smaller as confirmed by lower intensity of the 14 kDa protein band. The 20 kDa protein appears as a result of *Flavobacterium psychrophilum* infection [29] or is a thermal shock protein secreted in response to an increase in ambient (water) temperature.

The presence of lymphatic system cells and specific proteins indicated that at the time of the sample collection, the fish experienced stress associated with an environmental change and were most probably affected by a pathogenic agent unidentified in this study. The histological staining, cytological images and protein separation demonstrated that the seasons of the year (spring–summer and autumn–winter periods) significantly affect the condition of Carpathian barbel skin, which may limit the use of this species as a bioindicator. However, the analysis of mucus protein composition and its changes caused by external environment factors, including stress, are claimed to be an effective tool for monitoring alterations in the aquatic environment [30].

The skin, as an organ in constant contact with the surrounding environment, is subject to many morphological and physiological changes resulting from environmental stresses [14]. These changes allow for monitoring both the animal condition and the influence of the environment on the indicator species. Assessment involving physical and chemical changes in mucus is in fact a noninvasive monitoring method. So far, the results of mucus protein electrophoresis have not been compared with morphological and histological changes in the common integument triggered by environmental factors.

A saprobic system, currently used in Poland to assess water quality, determines the degree of organic pollution. The aim of a biological analysis is to identify biocenosis composition in polluted waters, and to pinpoint qualitative and quantitative changes in this composition caused by specific waste water. The system involves also fish as bioindicators. A saprobic index is calculated based on the tolerance of the indicator species with a specific saprobic value [2,31].

The system is, however, far from perfect, as the results of the ecosystem analysis depend on many factors, such as the abundance of indicator species, the need to test samples of various water body zones (seston, bentos and periphyton), the inability to analyze all types of pollution or the use of bioindicators to examine water of different geographical zones. Moreover, the methods for assessing the condition of rivers vary greatly in individual countries [11,12]. The World Health Organization (WHO) recommends estimation of water conditions in Europe based on the presence of fish highly sensitive to pollution (e.g., brown trout (*Salmo trutta*, Salmonidae), roach (*Rutilus rutilus*, Cyprinidae), zander (*Sander lucioperca*, Percidae) and moderately sensitive to pollution (e.g., perch (*Perca fluviatilis*, Percidae), carp (*Cyprinus carpio*, Cyprinidae) or bleak (*Alburnus* spp., Cyprinidae) [11].

Fish used as bioindicators must be kept under the investigated conditions for a specified period of time to assess their tolerance to water pollution, as well as changes in their morphology, physiology and behavior. A comprehensive and complete assessment of the environmental condition and functioning should involve simultaneous use of several independent methods. Barbel, a fish increasingly often found in Polish rivers, lakes and fishing ponds, is one of the species used in water quality monitoring [11,32].

In its natural environment, the Carpathian barbel usually lives in fast-flowing watercourses with a hard bottom. The species range is very wide and reaches up to the middle parts of lowland rivers. The Carpathian barbel inhabits fresh waters of southern Asia, Africa and Europe. Most species occur in Southeast Asia, and in Europe they can be found in the basins of the Loire, the Rhone, the Rhine, the Danube, the Elbe, the Oder, the Vistula, the Thames, the Nemunas, the Dniester, the Dnieper, and Iberian Peninsula rivers [33]. The widespread distribution of this species in fresh waters makes it a popular bioindicator in various countries. Other representatives of the Barbus genus can also be used as bioindicators.

## 5. Conclusions

Carpathian barbel, similar to other fish species, is frequently used as a bioindicator of natural environmental pollution. Seasonal changes in the water environment influences both skin morphology and physiology (quality and quantity of subsequent skin cells, mucus protein composition and cytological picture).

While monitoring the condition of water in reservoirs (ponds and lakes) and watercourses (rivers and streams), it is important to distinguish between changes caused by chemical and biological pollution and cyclical modifications of the fish’s common integument that occur throughout the year.

## Figures and Tables

**Table 1:** Composition of the stacking (2) and separating (1) gels.

	AA/BA 30:0.8	Separating Gel Buffer	Distilled Water 100 mg/ml	APS	TEMED	Gel Volume
Gel 1	3.25 mL	2.5 mL	4.21	30 mL	10 mL	10 mL
Gel 2	0.65 mL	1.25 mL	3.1	30 mL	7 mL	5 mL

**Table 2:** Qualitative and quantitative changes in mucus proteins depending on the season.

Season	Band	Weight (kDa)
Spring-summer	++	67
	++	20
	++	14
Autumn-winter	++	90
	++	60
	+	30
	+	14

## References

[1] Bohác J., Fuchs R., Jeffery D.W., Madden B. (1991). The Structure of Animal Communities as Bioindicators of Landscape Deterioration. Bioindicators and Environmental Management.

[2] Zimny H. (2006). Ekologiczna Ocena Stanu Srodowiska. Bioindykacja i Biomonitoring.

[3] Gadzała-Kopciuch R., Berecka B., Bartoszewicz J., Buszewski B. (2004). Some considerations about bioindicators in environmental monitoring. Pol. J. Environ. Stud..

[4] Parmar T.K., Rawtani D., Agrawal Y.K. (2016). Bioindicators: The natural indicator of environmental pollution. Front. Life Sci..

[5] Kotlik P., Berrebi P. (2002). Genetic subdivision and biogeography of the Danubian rheophilic barb Barbus petenyi inferred from phylogenetic analysis of mitochondrial DNA variation. Mol. Phylogenet. Evol..

[6] Kotlik P., Tsigenopoulos C.S., Ráb P., Berrebi P. (2002). Two new Barbus species from the Danube river basin, with redescription of B. petenyi (Teleostei: Cyprinidae). Folia Zool. Praha.

[7] Reichhol J., Steinbach G., Militz C. (1994). Wielka Encyklopedia Ryb: Słodkowodne i Morskie Ryby Europy.

[8] Terofal F., Militz C. (1997). Ryby słodkowodne. Leksykon przyrodniczy.

[9] Nilsson G.E. (2007). Gill remodeling in fish–A new fashion or an ancient secret?. J. Exp. Biol..

[10] Schaack S., Chapman L.J. (2003). Interdemic variation in the African cyprinid Barbus neumayeri: Correlations among hypoxia, morphology, and feeding performance. Can. J. Zool..

[11] Jankowski W. (1994). Zastosowanie Bioindykacji w Praktyce Monitoringu Srodowiska na Przykładzie Północno-Wschodniej Polski.

[12] Sadowska U. (2012). Ranga bioindykacji w ekotoksykologii wód. Studia Ecol. Bioeth..

[13] Hawkes J.W. (1974). The structure of fish skin. Cell Tissue Res..

[14] Vernerey F.J., Barthelat F. (2014). Skin and scales of teleost fish: Simple structure but high performance and multiple functions. J. Mech. Phys. Solids.

[15] Ellis R.E., Yuan J.Y., Horvitz H.R. (1991). Mechanisms and functions of cell death. Annu. Rev. Cell Dev. Biol..

[16] Fuchs E., Segre J.A. (2000). Stem cells: A new lease on life. Cell.

[17] Witkowski A., Kaleta K., Kuryszko J., Kusznierz J. (2004). Histological structure of the skin of Arctic Charr, Salvelinus alpinus (L) from Spitsbergen. Acta Ichthyol. Piscat..

[18] Iwama G.K., Thomas P.T., Forsyth R.B., Vijaya M.M. (1998). Heat Shock Protein Expression. Rev. Fish Biol. Fish..

[19] Cho W.J., Cha S.J., Do J.W., Choi J.Y., Lee J.Y., Jeong C.S., Cho K.J., Choi W.S., Kang H.S., Kim H.D. (1997). Novel 90-kDa Stress Protein Induced in Fish Cells by Fish Rhabdovirus Infecton. Biochem. Biophys. Res. Commun..

[20] Belles C., Kuhl A., Nosheny R., Cardin S.R. (1999). Plasma Membrane Expression of Heat Shock Protein 60 In Vivo in Response to Infecton. Infect. Immun..

[21] Sarge K.D., Murphy S.P., Morimoto R.I. (1993). Activation of heat shock gene transcription by heat shock factor 1 involves oligomerization, acquisition of DNA-binding activity, and nuclear localization and can occur in the absence of stress. Mol. Cell Biol..

[22] Sussman-Turner C., Renfro J.L. (1995). Heat-shock-stimulated transepithelial daunomycin secretion by pounder renal proximal tubule primary cultures. Am. J. Physiol. Cell Physiol..

[23] Sanders B.M., Martin L.S. (1993). Stress proteins as biomarkers of contaminant exposure in archived environmental samples. Sci. Total Environ..

[24] Subjeck J.R., Sciandra J.J., Johnson R.J. (1982). Heat shock proteins and thermotolerance; a comparison of induction kinetics. Br. J. Radiol..

[25] Udelsman R., Blake M.J., Stagg C.A., Li D., Putney D.J., Holbrook N. (1993). Vascular heat shock protein expression in response to stress. J. Clin. Invest..

[26] Udelsman R., Li D., Stagg C.A., Gordon C.B., Kvetnasky R. (1994). Adrenergic regulation of adrenal and aortic heat shock protein. Surgery.

[27] Udelsman R., Blake M.J., Stagg C.A., Holbrook N. (1994). Endocrine control of stress-induced heat shock protein 70 in vivo. Surgery.

[28] Misra S., Zafarullah M., Price-Haughey J., Gedamu L. (1989). Analysis of stress-induced gene expression in fish cell lines exposed to heavy metals and heat shock. Biochim. Biophys. Acta.

[29] Massias B., Dumetz F.M., Urdaci C., Le Hénaf M. (2004). Identification of P18, a surface protein produced by the fish pathogen Flavobacterium psychrophilum. J. Appl. Microbiol..

[30] Sanders B.M. (1993). Stress proteins in aquatic organisms: An environmental perspective. Crit. Rev. Toxicol..

[31] Zahradkova S., Soldan T., Jørgensen S.E., Fath B.D. (2008). Ecological indicators/Saprobic system. Encyclopedia of Ecology.

[32] Authman M.M.N., Zaki M.S., Khallaf E.A., Abbas H.H. (2015). Use of fish as bio-indicator of the effects of heavy metals pollution. J. Aquac. Res. Dev..

[33] Bogutskaya W. (2004). On Barbus waleckii Rolik, 1970 (Cyprinidae) in Ukraine with brief remarks on species from the genus Barbus Cuvier, 1816, distributed in the Dniestr and Vistula drainages. Vestn. Zool..

